# Effect of enamel surface treatment via Er, Cr: YSGG laser and nano-hydroxyapatite toothpaste on mineral content of primary teeth via x-ray diffractometer: an in-vitro study

**DOI:** 10.1038/s41405-026-00418-z

**Published:** 2026-04-11

**Authors:** Fatma Mohamed Elhussini, Dina Hamdy, Ahmed Hammad Abodouh, Gehan Gaber Allam

**Affiliations:** 1 Faculty of Dentistry, Future University in Egypt, 2016. MSc student at the Pediatric Dentistry and Dental Public Health Department, Faculty of Dentistry, Ain Shams University, Cairo, Egypt; 2 Associate Professor at the Pediatric Dentistry and Dental Public Health Department, Faculty of Dentistry, Ain Shams University, Cairo, Egypt; 3 Physics Department, Faculty of Science, Al-Azhar University, Cairo, Egypt; 4 Professor at the Pediatric Dentistry and Dental Public Health Department, Faculty of Dentistry, Ain Shams University, Cairo, Egypt

**Keywords:** Paediatric dentistry, Bisphosphonates in dentistry

## Abstract

**Purpose:**

This study evaluated the effect of Er,Cr:YSGG laser treatment, with or without nano-hydroxyapatite (nHAP) toothpaste, on the mineral content of primary enamel specimens, with emphasis on their remineralization potential in pediatric dentistry.

**Materials and methods:**

This in vitro study included 33 sound primary anterior teeth, from which enamel slabs were prepared and subjected to demineralization for 96 hours. Specimens were randomly assigned to three groups (*n* = 11): Group A (nHAP toothpaste), Group B (Er,Cr:YSGG laser, 2780 nm), and Group C (combined laser and nHAP toothpaste). Mineral phase composition was assessed using X-ray diffraction (XRD).

**Results:**

Quantitative phase analysis revealed an increase in apatite crystal content in all groups following treatment. Group C showed the highest increase (7.4%) and exhibited a complete transformation into apatite structures. Groups A and B demonstrated comparable increases (5.1% and 4.8%, respectively), with no statistically significant difference between them.

**Conclusions:**

Both Er,Cr:YSGG laser irradiation and nHAP toothpaste enhanced apatite formation in demineralized primary enamel; however, their combined application produced the most pronounced remineralization effect.

## Introduction

Dental caries is a common chronic disease globally and poses a major public health issue, particularly for children. Despite advances in oral healthcare and preventive measures, a significant number of children globally are still affected by early childhood caries, impacting their nutrition, speech development, growth, and overall quality of life (QOL) [1].

The initiation and progression of dental caries arise from a complicated interaction among cariogenic microorganisms, fermentable dietary carbohydrates, and susceptible tooth structures. Acidogenic bacteria, particularly Streptococcus mutans, metabolize carbohydrates to produce organic acids such as lactic acid, leading to a localized drop in plaque pH. When the pH declines below the critical threshold of 5.5, enamel hydroxyapatite (HAP) crystals undergo dissolution, initiating the demineralization process [2].

For an extended period, fluoride has been the gold standard for cavity prevention. This is owing to its potential to improve enamel resistance to acid dissolution by forming fluorapatite (FAP) and promoting remineralization of the enamel surface. However, the administration of fluoride-containing products in young children has raised concerns owing to the risk of dental fluorosis caused by inadvertent ingestion during toothbrushing [3].

These limitations have led to increased interest in biomimetic remineralizing agents that can restore the mineral content of enamel, like natural processes. Nano-HAP (nHAP) has garnered significant attention owing to its striking resemblance to the natural mineral structure of enamel and dentin. nHAP can penetrate demineralized enamel as a result of its nanoscale particle size, bind to HAP rods, and act as a template for new crystal formation, thereby promoting deeper remineralization [4].

In parallel, laser technology has emerged as an adjunctive tool in preventive dentistry. Lasers from the erbium family, particularly the Er, Cr: YSGG type operating at a wavelength of 2780 nm, can impactively ablate dental hard tissues through micro-explosive events resulting from the rapid heating and vaporization of water molecules within the enamel structure [5].

While both nHAP toothpaste and Er,Cr:YSGG laser therapy have independently demonstrated benefits in preventing demineralization and enhancing enamel remineralization, there is limited evidence regarding their combined impact, especially in primary teeth [6].

This study evaluated the effect of Er,Cr:YSGG laser treatment, with or without nHAP toothpaste, on the mineral content of primary enamel. The null hypothesis was that there is no statistically significant difference between Er,Cr:YSGG laser irradiation, nHAP toothpaste and the combination of them on the mineral content of primary teeth.

## Study setting

This in vitro investigation was done at the Faculty of Dentistry, Ain Shams University, in collaboration with the Department of Pediatric Dentistry and Dental Public Health, as well as the Nanotechnology Research Center at the British University in Cairo.

## Ethical approval

The existing work was conducted in February 2022 following an exemption granted by the Research Ethics Committee of the Faculty of Dentistry, Ain Shams University (FDASU-REC), Cairo, Egypt. Ethical approval for the use of extracted teeth in the research was obtained under approval code FDASU-RECEM022204. This study was conducted in accordance with the Declaration of Helsinki.

## Study design

An in vitro experimental study

## Materials and methods

### Sample size calculation

The sample size determination was performed to achieve adequate statistical power for testing the null hypothesis that no significant difference exists in the remineralization potential, based on mineral content, between nHAP and Er, Cr: YSGG laser treatment in demineralized primary anterior teeth. Setting the significance level (α) at 0.05 and the power (1–β) at 80% (β = 0.2), with an effect size (f) of 0.582 obtained from prior research [7]. The minimum required sample size was calculated to be 33 specimens, corresponding to 11 samples per experimental group. The calculation was carried out via G*Power software, version 3.1.9.7 [8].

### Teeth selection

Thirty-three extracted human primary anterior teeth, obtained following regular exfoliation or extraction for over-retention, were collected from the Department of Pediatric Dentistry and Dental Public Health, Faculty of Dentistry, Ain Shams University.

Inclusion criteria: Freshly extracted, sound teeth.

Exclusion criteria: Teeth were inspected under a stereomicroscope, and those with caries, cracks, restoration, and hypoplasia were excluded.

### Sample preparation and storage

All extracted teeth were thoroughly cleaned, polished, and subsequently stored in normal saline solution at 4 °C for a period not exceeding one month [9]. The crowns were carefully separated from the roots at the cemento-enamel junction via a straight fissure carbide bur mounted on a high-speed, water-cooled handpiece [10].

Enamel slabs measuring 4 × 4 × 2 mm³ were sectioned from the collected teeth via diamond discs [11]. To remove residual surface debris, the prepared specimens were immersed in 50% ethanol for 10 minutes, followed by gentle air drying under ambient conditions [12].

### Demineralization stage

Each enamel slab was then immersed in a standardized demineralizing medium composed of 0.05 M acetic acid, 2.2 mM CaCl₂, and 2.2 mM NaH₂PO₄, with the solution pH adjusted to 4.4 using 1 M KOH. The demineralization process was maintained for 96 hours under controlled conditions [13]. Following demineralization, specimens were rinsed in distilled water under stirring for 10 minutes, followed by air-drying [14].

### Sample grouping and randomization

Following demineralization, the specimens were randomly distributed into three experimental groups (*n* = 11 per group) using a simple randomization table generated in Microsoft Excel 2007 [11]. Each group received a distinct treatment modality as follows: Group A: nHAP toothpaste (APAGARD M-Plus, SANGI Co., Ltd., Japan). Group B: Er, Cr: YSGG laser (2780 nm; Waterlase, Biolase, USA). Group C: Er, Cr: YSGG laser followed immediately by application of nHAP toothpaste

To facilitate manipulation during testing, each enamel specimen was mounted in a self-cured acrylic resin block within a circular plastic mold. To preserve methodological reliability and prevent potential bias, a double-blind design was implemented; neither the statisticians nor the investigators responsible for the experimental procedures, measurements, or calibrations were aware of the treatment assignments.

### Toothpaste treatment

Applications were performed at 0, 8, 24, and 32 hours. Between applications, all enamel specimens were immersed in freshly prepared artificial saliva formulated to simulate the oral environment and sustain a physiologic pH of 7.0. The composition of this medium consisted of 14.4 mM NaCl, 16.1 mM KCl, 0.3 mM CaCl₂·6H₂O, 2.9 mM K₂HPO₄, 1.0 mM CaCl₂·2H₂O, and 0.10 g/100 ml sodium carboxymethylcellulose [15].

### Er, Cr: YSGG laser irradiation

Specimens allocated to Groups B and C were subjected to laser treatment using an Er,Cr:YSGG laser device (Waterlase, Biolase, USA) under the supervision of qualified academic staff at Al-Azhar University. The MZ6 sapphire tip (600 μm diameter, 9 mm length) was mounted according to the manufacturer’s instructions and examined microscopically at ×30 magnification (Viqsy, Japan) to verify the absence of surface damage, debris, or contamination prior to use. Throughout all irradiation procedures, the operator utilized OD 5+ protective eyewear designed for the specific wavelength of the laser to ensure safety compliance. The irradiation parameters were standardized as follows: 0.5 W output power, 60 μs pulse duration (H mode), 20 Hz repetition rate, and a cooling spray ratio of 60% water to 40% air, with a total exposure time of 15 seconds [16]. Prior to laser application, each specimen was removed from the artificial saliva, gently air-dried for three seconds to remove surface moisture, and immediately subjected to irradiation to minimize dehydration-related artifacts. During treatment, the laser handpiece was held perpendicular to the enamel surface at a consistent distance of 1–2 mm to maintain a uniform and reproducible energy distribution [17].

Samples were subjected to the following test immediately after demineralization (baseline) and after remineralization treatment for each group.

### X-ray diffraction (XRD) analysis

The degree of structural transformation resulting from each treatment protocol was assessed using X-ray diffraction (XRD) analysis. The relative peak intensity ratios were determined from the central region of each enamel specimen to quantify phase changes and crystal reorganization. Measurements were conducted using an Empyrean 3rd-generation X-ray diffractometer (Malvern Panalytical), operating with a CuKα radiation source (λ = 1.54 Å) at 40 kV and 30 mA under Bragg-Brentano geometry. The optical configuration of the diffractometer included a Soller slit (0.04 rad), a divergence slit (0.25 °), an anti-scattering slit (0.5 °), and a beam mask (5 mm). A proportional point detector was employed to record the diffracted beams, which were subsequently displayed as diffractograms plotting intensity (y-axis) against diffraction angle 2θ (x-axis). Scans were performed over a 2θ range of 5°–90°, with a step size of 0.02°, step time of 0.045 s, and scan speed of 0.0444°/s [18].

Data processing, including phase identification and quantitative assessment, was executed using High Score Plus software (Malvern Panalytical, Netherlands). Crystalline phases were matched to standard reference patterns obtained from the ICDD PDF-2 database (International Center for Diffraction Data, USA). Quantitative phase analysis (QPA) was performed utilizing the Rietveld refinement method to ensure precision and reproducibility. All instrumental and analytical conditions were carefully standardized and maintained constant before and after treatment to allow direct comparison between the tested groups [19].

### Statistical analysis

Data analysis was done via IBM SPSS Statistics version 27 (Chicago, IL, USA). The normality of the data distribution was verified through the Shapiro–Wilk test, along with a visual evaluation of histograms. For variables that did not meet parametric assumptions, results were expressed as medians with interquartile ranges (IQRs). Changes within individual groups over time were examined using the Wilcoxon signed-rank test, while comparisons among the three study groups were performed using the Kruskal–Wallis test and Mann Whitney-test to compare each group and post hoc (Bonferroni correction). A two-sided *P* < 0.05 was interpreted as statistically significant.

## Results

All samples within each group underwent identical treatment, and the middle-valued sample (median) result from each group was highlighted. The median (the middle value) was preferred as the data contained outliers. In this study, as we use natural human enamel samples, the ratios of certain phases (e.g., HAP) displayed considerable variation across samples, and some phases were present only in specific samples while absent in others. It’s known that the QPA method used in XRD has a predictable error of 2–3%, which limits its ability to detect phases present at low concentrations accurately. Consequently, the data was not symmetrically distributed and included outliers and skewness. Therefore, the median as the primary measure of central tendency was justified, as it minimizes the influence of extreme values and provides a more accurate and representative summary of the dataset.

**Fig. 1:**
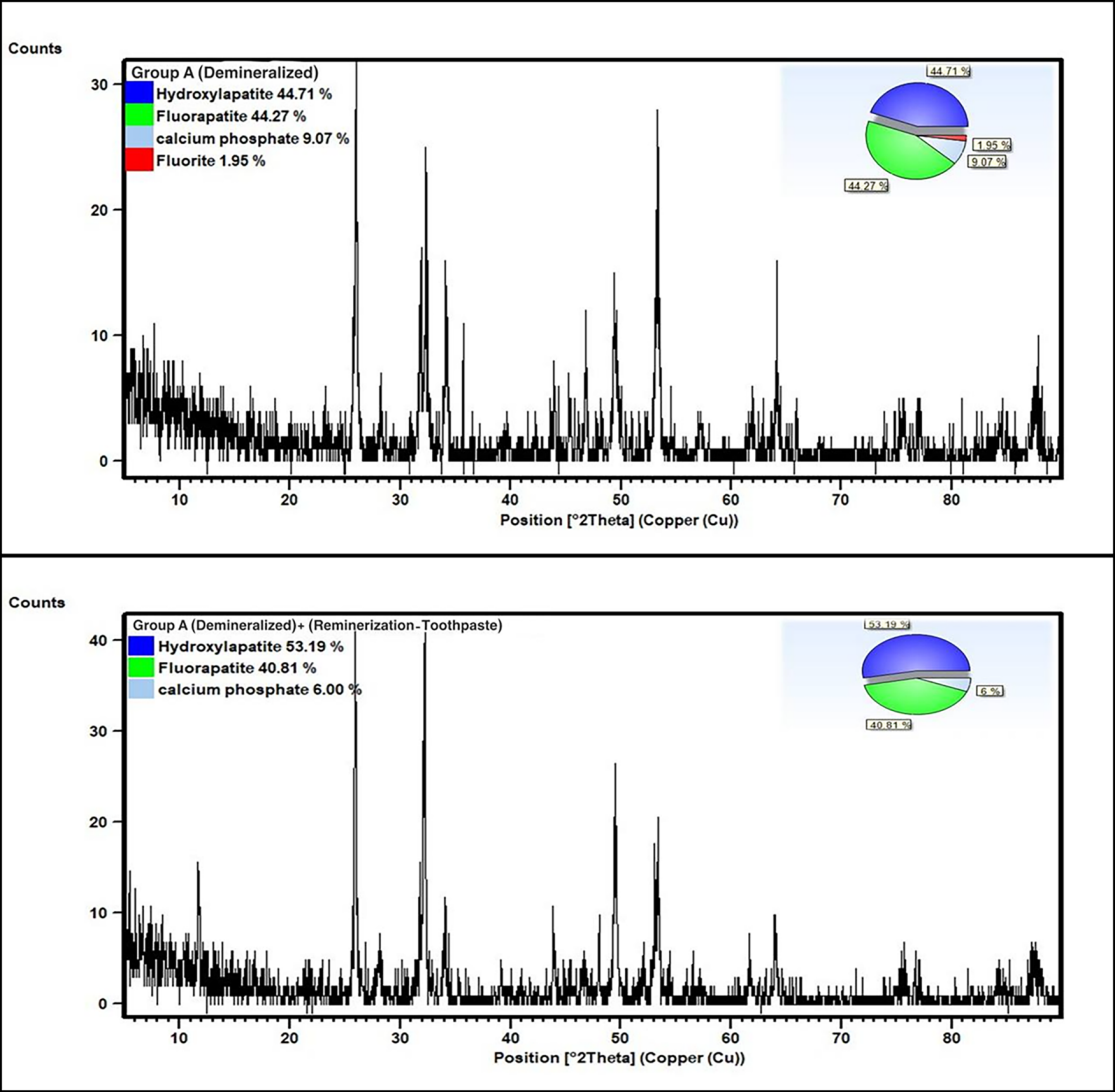
Group A demineralized and after remineralization. The upper panel represents demineralized enamel (Group A), while the lower panel shows the same group after treatment with remineralizing toothpaste. Diffraction intensity (counts) is plotted against the diffraction angle (2θ, Cu source). Insets display pie charts illustrating the relative percentages of identified phases: hydroxyapatite, fluorapatite, calcium phosphate, and fluorite. After remineralization, an increase in hydroxyapatite content and a reduction in calcium phosphate are observed, indicating improved mineral recovery.

In group A (median sample), the QPA exhibited 44.7% hydroxylapatite (HAP) (PDF No #00-064-0738), 44.2% FAP (PDF No #01-070-8136), 9.1% calcium phosphate (PDF No #01-070-0364), and 2% calcium fluoride (PDF No #00-048-1298) before remineralization. After toothpaste treatment, the ratios changed to 53.2% HAP, 40.8% FAP, and 6% Ca Phosphate. Table 1, Fig. 1

**Table 1:** Samples exhibiting an average increase in apatite crystal content per group.

(Group)	Before treatment		After treatment		Increase rate of apatite crystals (%)
	Phases ratios	Apatite crystals %	Phases ratios	Apatite crystals %	
(n-HAP Toothpaste)	44.7% HAP, 44.2% FAP, 9.1% Calcium Phosphate, and 2% Calcium Fluoride.	88.9%	53.2% HAP, 40.8% FAP, and 6.0% Calcium Phosphate.	94%	5.1%
(Er,Cr:YSGG laser)	49.4% HAP, 44.4% FAP, 0.8% Calcium Fluoride, and 5.3% Calcium Mg Phosphate	93.8%	59.6% HAP, 39.0% FAP, and 1.4% Calcium Mg Phosphate.	98.6%	4.8%
(laser + toothpaste)	46.1% HAP, 46.5% FAP, and 7.4%Calcium Phosphate.	92.6%	48.8% HAP, 51.2% FAP.	100%	7.4%

*HAP* hydroxylapatite, *FAP* fluorapatite.

**Fig. 2:**
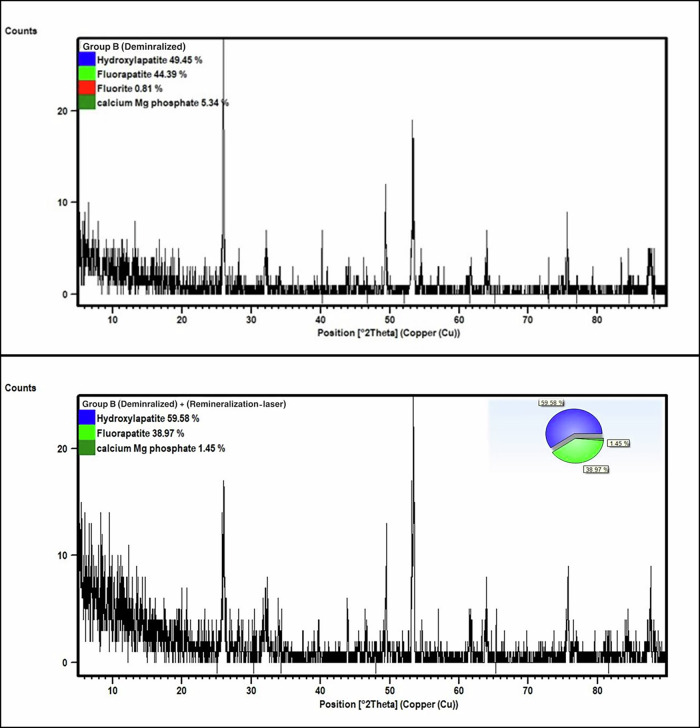
Group B demineralized and after remineralization. The upper panel represents demineralized enamel (Group B), while the lower panel shows the same group following laser treatment. Diffraction intensity (counts) is plotted as a function of diffraction angle (2θ, Cu radiation). Insets illustrate pie charts of the relative phase composition, including hydroxyapatite, fluorapatite, fluorite, and calcium magnesium phosphate. Following laser treatment, an increase in hydroxyapatite content and a reduction in calcium magnesium phosphate are observed, indicating enhanced remineralization and improved mineral structure.

In group B (median sample), QPA revealed 49.4% HAP, 44.4% FAP, 0.8% calcium fluoride, and 5.3% calcium Mg phosphate (PDF No #01-082-9075) before remineralization. After Laser treatment, the ratios became 59.6% HAP, 39.0%, 3.8 FAP, and 1.4% calcium Mg phosphate. Table 1, Fig. 2

**Fig. 3:**
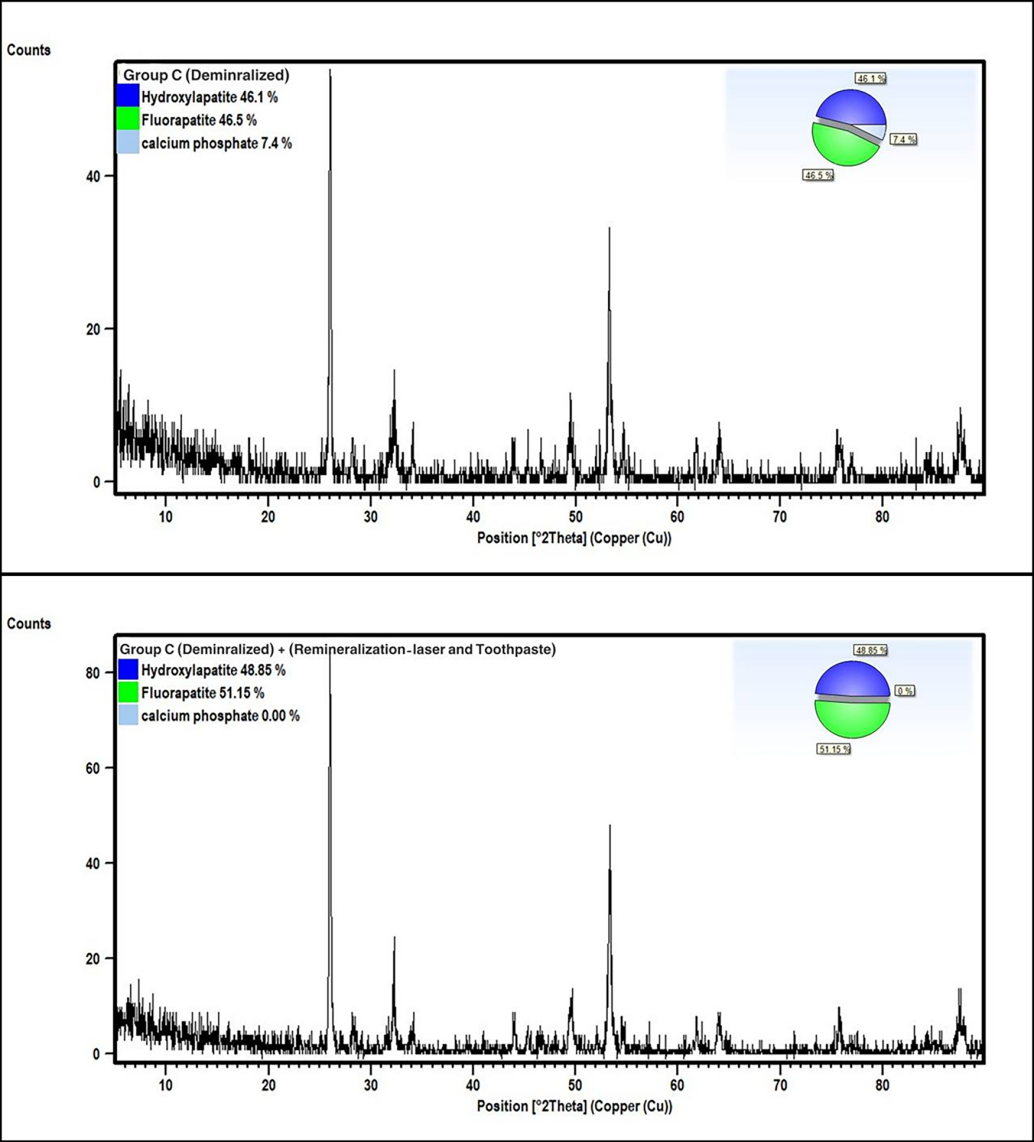
Group C demineralized and after remineralization. The upper panel represents demineralized enamel (Group C), while the lower panel shows the same group following the combined treatment (laser and toothpaste). Diffraction intensity (counts) is plotted against the diffraction angle (2θ, Cu radiation). Insets present pie charts of the relative phase composition, including hydroxyapatite, fluorapatite, and calcium phosphate. After treatment, an increase in fluorapatite and hydroxyapatite content with the complete disappearance of calcium phosphate is observed, indicating enhanced remineralization and improved mineral phase stability.

In group C (median sample), QPA exhibited 46.1% HAP, 46.5% FAP, and 7.4% calcium phosphate before remineralization. After combined treatment (toothpaste and laser), the QPA revealed a composition of 48.8% HAP and 51.2% FAP, pointing to a complete transformation of the phases into apatite structures. Table 1, Fig. 3

**Fig. 4:**
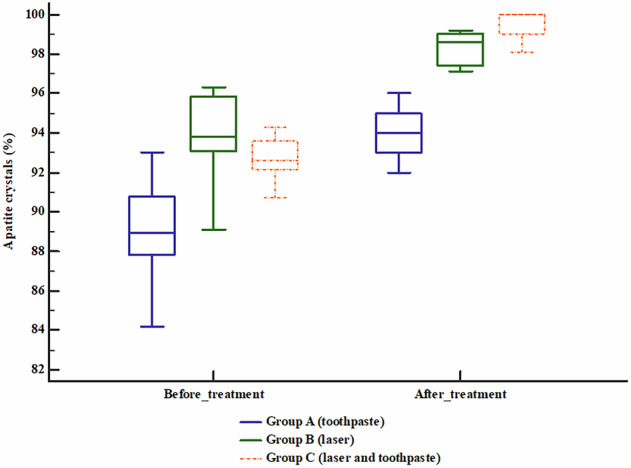
Box plot chart showing the apatite crystals of the studied groups. The central line within each box represents the median, the box indicates the interquartile range (IQR), and the whiskers denote the minimum and maximum values. All groups show an increase in apatite crystal percentage after treatment, with Group C demonstrating the highest post-treatment values, followed by Group B and Group A, indicating the superior effect of the combined treatment.

Apatite crystals were significantly increased after treatment compared with before treatment in groups A (toothpaste), B (laser), and C (laser and toothpaste) (*P* < 0.05). Table 2, Fig. 4

**Table 2:** Intragroup comparison of apatite crystals in the studied samples via the Wilcoxon test.

	Apatite crystals %		*P*
	Before remineralization	After remineralization	
Group A (toothpaste) (*n* = 11)	88.9 (87.9–90.5)	94 (93–95)	0.003*
Group B (laser) (*n* = 11)	93.8 (93.1–95.6)	98.6 (97.6–99)	0.003*
Group C (laser and toothpaste) (*n* = 11)	92.6 (92.2–93.25)	100 (99–100)	**0.003** ^*^

Data is presented as median (IQR) or mean ± SD.

*Significant as *P* < 0.05. Bold P-values reflect significant intragroup differences between measurements before and after remineralization within each group.

**Fig. 5:**
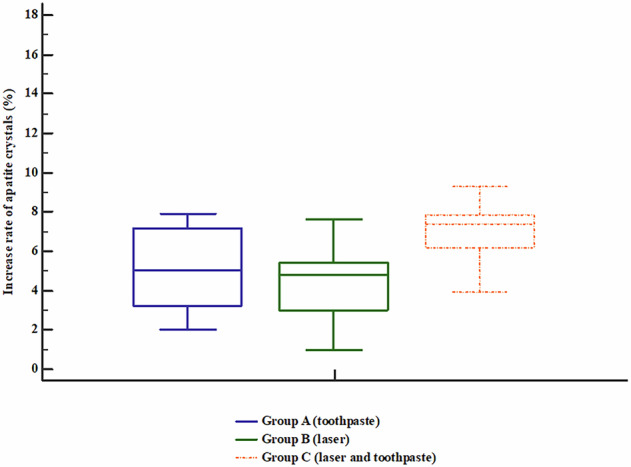
Box plot chart showing the Increase rate of apatite crystals of the studied groups. The central line within each box represents the median, the box boundaries indicate the interquartile range (IQR), and the whiskers show the minimum and maximum values. Group C demonstrates higher median values compared to Groups A and B, indicating a greater increase in apatite crystal formation with the combined treatment.

The highest increase in crystal density was observed in group C (7.4%), which was significantly higher compared with groups A and B, while groups A (5.1%) and B (4.8%) exhibited comparability, while Bonferroni correction revealed significant difference between group B (laser) and group C (laser and toothpaste) only with no significant difference between other groups. Table 3,Fig. 5

**Table 3:** Intergroup comparison of the increase rate of apatite crystals among the studied samples, Kruskal-Wallis test.

	Increase rate of apatite crystals (%)	*P*	Post hoc	Post hoc by Bonferroni correction
Group A (toothpaste) (*n* = 11)	5.1(3.5–7.1)	0.010*	**P**_**1**_ = 0.384	**P1** = 1
Group B (laser) (*n* = 11)	4.8(3.3–5.35)		**P**_**2**_ = 0.036*	**P2** = 0.108
Group C (laser and toothpaste) (*n* = 11)	7.4(6.25–7.8)		**P**_**3**_ = 0.003*	**P3** = 0.009*

Data is presented as median (IQR). P1: P between groups A (toothpaste) and B (laser), P2: P between groups A (toothpaste) and C (laser and toothpaste), P3: P between groups B (laser) and C (laser and toothpaste).

*: Significant as *P* < 0.05. Bold values denote statistically significant differences in intergroup comparisons of the increase rate of apatite crystals.

## Discussion

The present study was conducted to test the effectiveness of combining Er,Cr:YSGG laser irradiation with nHAP toothpaste in enhancing apatite formation in comparison to either modality alone.

The Er,Cr:YSGG laser (2780 nm) was specifically selected because erbium-family wavelengths exhibit high absorption in both water and hydroxyapatite, making them particularly suitable for modifying dental hard tissues and enhancing enamel mineral resistance. Unlike diode lasers, which are primarily absorbed by pigmented soft tissues, Er,Cr:YSGG is capable of inducing surface recrystallization and reducing enamel permeability under sub-ablative parameters, thereby promoting remineralization [16, 20].

In Group A (demineralized enamel treated with APAGARD M-Plus), the nano-hydroxyapatite (nHAP)-containing toothpaste enhanced enamel remineralization by increasing the relative proportion and crystallinity of biologically stable apatite phases rather than by inducing new phase transformations. Nanoparticle deposition effectively filled microporosities, and because nHAP shares the hexagonal crystal structure of native enamel hydroxyapatite (HAP), an increase in HAP content was observed [21].

X-ray diffraction (XRD) patterns revealed increased intensity and sharpening of characteristic HAP peaks at 2θ ≈ 25.86° and 32.18°, indicating enhanced crystallinity. Increased intensity of fluorapatite (FAP) peaks at 2θ ≈ 26.05° and 32.25° suggests partial fluoride incorporation into the apatite lattice. Conversely, the calcium phosphate peak at 2θ ≈ 34.16° decreased in intensity following treatment, reflecting a reduction in the less stable phase. A calcium fluoride signal at 2θ ≈ 47.01° appeared only at background levels, likely due to its very low concentration and the detection limits of XRD [22].

These results support the effectiveness of nHAP-based toothpaste in promoting remineralization through the growth and stabilization of apatite crystals on enamel surfaces. This came in agreement with Huang and co-authors [23] who reported that increasing nHAP concentration up to 10% markedly enhanced the remineralization impact, with a pronounced improvement observed between the 5% and 10% concentrations.

These results are consistent with Imran and co-authors [24] who showed that nHAP promotes mineralization in early carious lesions by incorporating it into the porous enamel structure that is formed during demineralization. This process increases mineral content and surface hardness. Importantly, the efficacy of the mineralization process was determined to be significantly influenced by the particle size of nHAP.

In Group B, Er,Cr:YSGG laser (2780 nm) irradiation also resulted in an increased apatite phase fraction. This effect may be attributed to the absorption of (Er,Cr:YSGG laser, 2780 nm) by surface water and enamel hydroxyl (–OH) groups, converting optical energy into rapid, localized heat. This short thermal pulse can induce transient surface melting (microsecond scale) followed by rapid recrystallization (laser-induced thermal modification). During cooling, apatite crystals re-nucleate and preferentially grow along thermodynamically favorable planes, producing preferred orientation rather than a new phase. This is supported by the XRD pattern of Group B, where the HAP reflection at 2θ ≈ 53.4° increased in intensity while the HAP peak at 2θ ≈ 25.9° decreased after treatment, consistent with re-oriented crystallites and plane-dependent intensity changes. The extremely high cooling rate also promotes crystallite fusion and reorganization into larger, more ordered apatite crystals, reducing porosity, microcracks, lattice defects, and entrapped impurities—thereby improving enamel crystallinity. In addition, laser heating may enhance fluoride diffusion toward the surface, supporting fluorapatite (FAP) formation and improved enamel resistance [25].

This was corroborated by Dehghani and co-authors [26] who revealed that the Er,Cr:YSGG laser (2780 nm) has a high absorption rate by enamel HAP crystals, thereby increasing the potential for enamel remineralization.

Similarly, Abdelhamid et al. [27] reported that the application of Er: Cr: YSGG laser significantly promoted the remineralization of enamel lesions compared to artificial saliva.

In Group C, where Er,Cr:YSGG laser treatment was combined with nano-hydroxyapatite (nHAP) toothpaste application, additional enhancements were observed compared with either modality alone. During recrystallization following partial laser-induced surface melting, nHAP particles and dicalcium phosphate supplied by the toothpaste act as effective nucleation sites, promoting organized and epitaxial growth of hydroxyapatite on the enamel surface. This synergistic interaction facilitates re-nucleation and crystal growth during rapid cooling, leading to a marked increase in apatite crystal content and improved crystallinity. Such findings are consistent with previous reports demonstrating that laser irradiation enhances the remineralizing potential of nHAP by creating favorable surface conditions for crystal deposition and maturation, resulting in superior enamel mineral recovery and structural reinforcement [6].

This was reinforced by Yilmaz and co-authors [16] reported that Er,Cr:YSGG laser enhanced mineral stability of primary enamel when used with remineralization protocols.

Similarly, Mahdi and co-authors [20] who employed XRD to evaluate the impact of remineralization. They found that the remineralization of primary enamel affected by white spot lesions was significantly improved by combining Er,Cr:YSGG laser irradiation at sub-ablative parameters with acidulated phosphate fluoride (APF) gel application.

This finding aligned with El-Haddad and co-authors [28] who found that either the application of nHAP-containing toothpaste or fluoridated toothpaste, augmented by diode laser, improves the process of enamel remineralization and strengthens its integrity.

Therefore, while both nHAP toothpaste and Er,Cr:YSGG laser independently contribute to enamel remineralization, the combined approach achieved the most substantial mineral recovery. Thus, the findings of the present study led to rejection of the null hypothesis.

The study was designed to evaluate the combined impact of Er,Cr:YSGG laser and nHAP toothpaste on the remineralization potential of primary enamel, representing an adjunctive approach for enhancing enamel remineralization for children. It was conducted via a well-controlled in-vitro design with standardized demineralization and remineralization protocols, ensuring high reproducibility and reliability of results. The use of QPA (XRD) allowed for comprehensive assessment of mineral content, providing both structural and functional insights.

Limitations: The in vitro conditions under which this investigation was conducted may not accurately replicate the complex oral environment, which is influenced by masticatory stresses, biofilm metabolism, and saliva flow. In addition, the evaluation was limited to short-term results, and there was no examination of the induced remineralization’s long-term persistence or mechanical durability. In addition, the contextualization of the findings within current clinical practice is restricted by the absence of a comparative arm that includes fluoride or other commonly employed remineralizing formulations. A negative control group could be added to future studies to improve understanding of the demineralization-remineralization process. Future studies integrating surface morphology are recommended.

Although powdering samples remain the most widely espoused preparation technique in X-ray diffraction (XRD) studies, measurements on solid samples are still acceptable and useable, mostly for investigating layered structures [29]. However, the present study was executed on tooth enamel and in this case, measurement of the preferred orientation of the solid sample is considered an advantage, as it exhibits slightly distinct properties depending on its specific direction of the enamel’s preferred orientation. In contrast, a powdered sample has a random orientation, which results in average properties that do not reflect the exact material’s true properties [30]. Thus, this method offers a more realistic assessment, as the samples are measured in essentially the same form in which they are used, reflecting their behavior under daily circumstances. Moreover, the study includes toothpaste treatment, which requires measuring the samples in their solid form (a non-destructive method) to allow repeated measurements after treatment, which would be difficult with powdered samples [31].

## Conclusion

The Er,Cr:YSGG laser and nHAP toothpaste combination provided the most effective remineralization strategy for demineralized primary enamel, producing the highest increase in apatite crystal content and the most significant transformation into stable apatite phases. While both nHAP and laser treatment alone demonstrated significant improvements, their impacts were comparable and less pronounced than those of the combined protocol.

## Supplementary information

Apatite Percentage Data

supplementary diffractograms

supplementary legends

## Data Availability

Data are available on reasonable request from corresponding author.
