# Supplementary material for: Effect of enamel surface treatment via Er, Cr: YSGG laser and nano-hydroxyapatite toothpaste on mineral content of primary teeth via x-ray diffractometer: an in-vitro study
Source: BDJ Open. 2026 Apr 11;12:34. doi: 10.1038/s41405-026-00418-z (PMC13070032; doi:10.1038/s41405-026-00418-z)
Supplement: Supplementary file 1 — Apatite Percentage Data [file 41405_2026_418_MOESM1_ESM.docx]

# Apatite Percentage Data

| Group | Apatite % Before Treatment | Apatite % After Treatment | Increase Rate of Apatite Crystals (%) |
| --- | --- | --- | --- |
| A | 84.2 | 92 | 7.8 |
| A | 87.8 | 95 | 7.2 |
| A | 91 | 96 | 5 |
| A | 90 | 93 | 3 |
| A | 87.1 | 95 | 7.9 |
| B | 89.1 | 94.3 | 5.2 |
| B | 93.8 | 98.6 | 4.8 |
| B | 96.1 | 97.2 | 1.1 |
| B | 94.2 | 98.6 | 4.4 |
| B | 93.1 | 98 | 4.9 |
| C | 90.7 | 100 | 9.3 |
| C | 82.1 | 98.9 | 16.8 |
| C | 92.1 | 100 | 7.9 |
| C | 94.3 | 100 | 5.7 |
| C | 94.2 | 98.1 | 3.9 |
