# Supplementary material for: Effect of enamel surface treatment via Er, Cr: YSGG laser and nano-hydroxyapatite toothpaste on mineral content of primary teeth via x-ray diffractometer: an in-vitro study
Source: BDJ Open. 2026 Apr 11;12:34. doi: 10.1038/s41405-026-00418-z (PMC13070032; doi:10.1038/s41405-026-00418-z)
Supplement: Supplementary file 3 — supplementary legends [file 41405_2026_418_MOESM3_ESM.docx]

**Supplementary files legends:**

**Table:** apatite percentage data (for 5 samples of each group).

**Figures**: supplementary diffractograms ( demineralized and after remineralization for 5 samples of each group).
